# Safety and clinical activity of durvalumab combined with tremelimumab in recurrent/metastatic head and neck squamous cell carcinoma: a multicenter phase I study

**DOI:** 10.1016/j.esmoop.2024.103646

**Published:** 2024-07-23

**Authors:** A. Algazi, K.P. Papadopoulos, F. Tsai, A.R. Hansen, N. Angra, M. Das, S. Sheth, L.L. Siu

**Affiliations:** 1 Head and Neck Medical Oncology Program, University of California, San Francisco; 2 Clinical Research, South Texas Accelerated Research Therapeutics (START), San Antonio; 3 Medical Oncology, HonorHealth Research and Innovation Institute, Scottsdale, USA; 4 Medical Oncology, Princess Margaret Cancer Centre, Toronto, Canada; 5 Oncology R&D, AstraZeneca, Gaithersburg; 6 Division of Oncology, University of North Carolina Lineberger Cancer Center, Chapel Hill, USA; 7 Division of Medical Oncology and Hematology, Department of Medicine, Princess Margaret Cancer Centre, Toronto, Canada

**Keywords:** head and neck squamous cell carcinoma, PD-L1 inhibitor, CTLA-4 inhibitor, immunotherapy

## Abstract

**Background:**

Programmed cell death protein 1 (PD-1) inhibitors prolong survival versus chemotherapy in recurrent/metastatic head and neck squamous cell carcinoma (R/M HNSCC), which often expresses cytotoxic T-lymphocyte-associated protein 4 (CTLA-4) and programmed cell death-ligand 1 (PD-L1), providing a rationale for combined PD-(L)1 and CTLA-4 blockade. We report a phase I, open-label study of the PD-L1 inhibitor durvalumab plus the CTLA-4 inhibitor tremelimumab (NCT02262741).

**Methods:**

In dose exploration, two cohorts of previously treated patients received durvalumab 10 mg/kg plus tremelimumab 3 mg/kg, or durvalumab 20 mg/kg plus tremelimumab 1 mg/kg, for up to 12 months. Dose expansion comprised two cohorts of previously untreated patients with R/M HNSCC having baseline PD-L1 tumor cell (TC) expression ≥25% and <25% and one cohort of immunotherapy-pretreated patients with any PD-L1 level. All received durvalumab 20 mg/kg plus tremelimumab 1 mg/kg, then durvalumab 10 mg/kg, for up to 12 months. The primary endpoint was safety. The secondary endpoints were objective response rate (ORR) by RECIST version 1.1, pharmacokinetics, pharmacodynamics, and immunogenicity.

**Results:**

A total of 71 patients were treated. The median duration of exposure was 13.6 weeks for durvalumab and 13.1 weeks for tremelimumab. In dose exploration, no dose-limiting toxicities occurred. No maximum tolerated dose was identified. Treatment-related adverse events (TRAEs) occurred in 69.0% of patients; grade 3/4 and serious TRAEs occurred in 31.0% and 18.3%, respectively. TRAEs led to discontinuation in 9.9%. There were no treatment-related deaths. The ORR was 5.6% (95% confidence interval 1.6-13.8), including one complete response and three partial responses, all patients were in dose expansion with PD-L1 TC ≥25% and no prior immunotherapy exposure; three had ongoing responses ≥12 months. The median overall survival in the total population was 8.6 months. Soluble PD-L1 suppression was almost complete in all cohorts, suggesting target engagement. CD4+Ki67+ T cells were significantly elevated in all dose-expansion cohorts.

**Conclusions:**

Treatment was well tolerated. However, response rates were low despite target engagement, no drug–drug interactions, and no drug-neutralizing antibodies to durvalumab.

## Highlights

• Combination immunotherapy-based strategies are an active area of investigation in patients with R/M HNSCC.
• In this phase I study, durvalumab, a PD-L1 inhibitor, plus tremelimumab, a CTLA-4 inhibitor, was well tolerated and safe.
• This combination did not improve outcomes including overall survival versus historical data on anti-PD-(L)1 monotherapy.

## Introduction

Before 2019, the first-line therapy for unresectable, recurrent/metastatic (R/M) head and neck squamous cell carcinoma (HNSCC) was a combination of carboplatin or cisplatin, 5-fluorouracil, and cetuximab (the EXTREME regimen).^1^ This therapy provided a median overall survival (OS) of ∼10 months, but with a >80% incidence of grade ≥3 toxicity.^1^ In KEYNOTE 048, immunotherapy with pembrolizumab, an anti-programmed cell death protein 1 (PD-1) monoclonal antibody (mAb), as first-line treatment with or without chemotherapy, improved OS compared with EXTREME; however, long-term remission is seen in <20% of patients.^2^ Several trials are evaluating combinations of immune modulators to improve long-term survival.^3^

Combinations of mAbs against PD-1 or its ligand [programmed cell death-ligand 1 (PD-L1)] with anticytotoxic T-lymphocyte-associated protein 4 (CTLA-4) mAbs have shown promise across a range of advanced solid tumors, including small-cell lung cancer,^4^ urothelial carcinoma,^5^ renal cell carcinoma,^6^ melanoma,^7^ non-small-cell lung cancer (NSCLC),^8^ and hepatocellular carcinoma.^9^ Durvalumab targets PD-L1 and has demonstrated clinical activity in patients with HNSCC and PD-L1 tumor cell (TC) expression ≥25%.^10^ Tremelimumab, a human immunoglobulin G2 mAb that binds selectively to CTLA-4, has shown an acceptable safety profile and clinical activity across several tumor types.^11^ Durvalumab plus a fixed dose of tremelimumab [1 mg/kg every 4 weeks (Q4W)] for R/M HNSCC produced an objective response rate (ORR) of 7.8% among patients with PD-L1 TC <25% in the CONDOR study.^12^ There was no survival improvement versus durvalumab monotherapy in a PD-(L)1 inhibitor-naive population in the EAGLE study.^13^

Here we report results from a phase I study of durvalumab combined with tremelimumab in treatment-naive or previously treated patients [including those refractory to PD-(L)1 inhibitors] with R/M HNSCC (ClinicalTrials.gov NCT02262741).

## Methods

### Study design and patients

Patients in this multicenter, open-label, dose-exploration, and dose-expansion study had histologically or cytologically confirmed R/M HNSCC, with tumors in the oral cavity, oropharynx, hypopharynx, or larynx that were incurable by local therapy.

Patients in the dose-exploration phase had progressive disease with up to three prior treatment regimens for R/M disease. Patients who refused or were ineligible for standard approved therapy for R/M disease were permitted to enroll. Patients were eligible regardless of PD-L1 TC expression but were excluded if they had prior treatment with immune-mediating therapies.

The dose-expansion phase included three cohorts: patients who were previously untreated in the R/M setting with baseline PD-L1 TC ≥25%, patients with previously untreated R/M disease with baseline PD-L1 TC <25%, and patients with immunotherapy-pretreated R/M disease with any PD-L1 expression level. Patients were eligible for the PD-L1 TC ≥25% and PD-L1 TC <25% cohorts if they had refused or were ineligible for standard therapies. Patients eligible for the immunotherapy-pretreated cohort had documented disease progression on anti-PD-(L)1 monotherapy in the R/M setting.

In all cohorts, systemic therapy as part of induction, chemoradiotherapy, or adjuvant treatment was allowed in the curative setting. Additional eligibility criteria are listed in the Supplementary Materials, available at https://doi.org/10.1016/j.esmoop.2024.103646.

All patients provided written informed consent. The study protocol was approved by the institutional review board or ethics committee for each center, and the study was conducted in accordance with the Declaration of Helsinki and the International Council for Harmonisation guidelines on Good Clinical Practice, as well as applicable local laws and requirements.

### Study treatment

The dose-exploration phase included two cohorts treated for up to 12 months: (i) durvalumab 10 mg/kg intravenously (IV) every 2 weeks (Q2W) for up to 26 doses, plus tremelimumab 3 mg/kg IV Q4W for seven doses, and then every 12 weeks (Q12W) for two further doses; and (ii) durvalumab 20 mg/kg IV Q4W for up to 13 doses, plus tremelimumab 1 mg/kg IV Q4W for seven doses, and then Q12W for two doses (Supplementary Figure S1, available at https://doi.org/10.1016/j.esmoop.2024.103646).

All three cohorts in the dose-expansion phase received durvalumab 20 mg/kg plus tremelimumab 1 mg/kg Q4W for up to four doses each, followed by durvalumab alone 10 mg/kg Q2W to complete 12 months of treatment. Selection of these doses was informed by results from the dose-exploration phase and was primarily based on emerging safety data from ongoing studies at the time, including a phase Ib study in NSCLC, in which durvalumab 20 mg/kg plus tremelimumab 3 mg/kg was associated with dose-limiting toxicity (DLT; as defined in the Supplementary Materials, available at https://doi.org/10.1016/j.esmoop.2024.103646). The optimal regimen was determined to be durvalumab 20 mg/kg plus tremelimumab 1 mg/kg.^14^

### Safety and efficacy assessments

Patients were regularly assessed for safety at baseline, throughout treatment, and until 90 days after the end of treatment, and adverse events (AEs) were graded according to the National Cancer Institute’s Common Terminology Criteria for Adverse Events, version 4.03 (see Supplementary Materials, available at https://doi.org/10.1016/j.esmoop.2024.103646 for DLT definition).

Tumors were assessed at baseline according to RECIST, version 1.1^15^ (including evaluation for brain metastases) every 8 weeks on treatment, at the end of treatment, then every 3 months for 12 months after treatment, and every 6 months thereafter until the end of the study. Patients were followed for survival until the end of the study.

### Assessment of human papillomavirus status

Human papillomavirus (HPV) status was determined using archived and/or fresh tumor tissue, either by p16 immunohistochemistry or by HPV *in situ* hybridization.

### Assessment of PD-L1 expression

Fresh tumor biopsies were obtained at baseline from all patients and assayed centrally for expression of PD-L1 on TCs, using the VENTANA PD-L1 (SP263) immunohistochemistry assay (Ventana Medical Systems, Tucson, AZ).^16^ PD-L1 expression was measured as the percentage of TCs with membranes staining positive for PD-L1 at any intensity, and samples were classified as PD-L1 TC expression of ≥25% or <25%.

### Immunogenicity

Antidrug immunogenicity was evaluated in samples from patients who received one or more doses of durvalumab or tremelimumab and provided one or more post-treatment samples. Results were analyzed descriptively by summarizing the number and percentage of patients with detectable antidrug antibodies (ADAs) against durvalumab or tremelimumab.

### Pharmacokinetics and pharmacodynamics

Pharmacokinetics was evaluated in samples from patients who received one or more full doses of durvalumab or tremelimumab and provided one or more post-treatment samples. Individual durvalumab and tremelimumab concentrations were tabulated by treatment group along with descriptive statistics. No formal noncompartmental analysis was conducted due to the sparse pharmacokinetic sampling scheme. Pharmacodynamic analyses included soluble PD-L1 levels before and after treatment with durvalumab and/or tremelimumab to evaluate target engagement.

### Peripheral blood immunophenotyping

Two flow cytometry assays (T, B, and natural killer cell, and proliferating T cell) were designed and analytically validated to evaluate quantities and proliferation states of circulating lymphocyte populations following treatment in the exploration and expansion phases. Peripheral blood samples were collected at screening; predose days 1, 8, and 15; and predose thereafter on days 29, 57, 85, 113, and 169.

### Endpoints

The primary endpoint was safety. The secondary endpoints were efficacy [ORR, disease control, duration of response (DoR), progression-free survival (PFS) based on RECIST version 1.1, and OS], pharmacokinetics, and pharmacodynamics. T-cell bioanalysis was an exploratory endpoint.

### Statistical analyses

The planned sample size was 6-12 patients for the dose-exploration phase. The dose-expansion phase allowed for up to 60 patients with ∼20 patients per cohort. The population for DLT analysis included all patients enrolled in the dose-exploration phase who were treated with durvalumab and tremelimumab and completed safety follow-up for the DLT evaluation period or who had a DLT in this period. The time frame was defined as the period from the first dose of study drugs to the planned administration of the third dose of durvalumab and the second dose of tremelimumab. Other analyses included all patients who received at least one dose of any study drug (as-treated population). The ORR and disease control rate were estimated with 95% confidence intervals (CIs), using exact binomial distribution. Time-to-event analyses for DoR, PFS, and OS were determined with the Kaplan–Meier method.

## Results

### Patients and treatment exposure

Between October 2014 and September 2017, 71 patients were included in the as-treated population. The median age was 63.0 years (range 34-90) years. Most patients were male (81.7%), had an Eastern Cooperative Oncology Group performance status of 1 (69.0%), and were current or former smokers (54.9%; Supplementary Table S1, available at https://doi.org/10.1016/j.esmoop.2024.103646). HPV status was positive in 29 of the 71 (40.8%) patients tested. Of the 66 patients with known PD-L1 status, 25 (37.9%) had PD-L1 TC ≥25% and 41 (62.1%) had PD-L1 TC <25%. Overall, 38 of the 66 (57.6%) patients had no prior treatment for R/M disease. In the immunotherapy-pretreated cohort of the expansion phase, most patients had received either two (60.0%) or three (35.0%) prior lines of therapy. At the time of database lock (8 November 2017), the median duration of follow-up for the total population was 6.9 months (range 0.3-24.4 months).

Overall, the median duration of exposure to durvalumab was 13.6 weeks (range 1.1-52.3) weeks, with a median of 3.5 (range 1-22) doses. The median exposure to tremelimumab was 13.1 weeks (range 1.1-32.0) weeks, with a median of 3.0 (range 1-8) doses.

In total, 62 (87.3%) patients discontinued treatment: 50 (70.4%) patients discontinued due to death (caused by disease under investigation in 46 patients, acute respiratory failure unrelated to treatment in 1 patient, sudden death in 1 patient, cardiopulmonary arrest in 1 patient, and unknown reasons in 1 patient); 11 (15.5%) patients discontinued due to withdrawal of consent, and 1 (1.4%) due to other reasons. Treatment was ongoing in nine (12.7%) patients. There were no differences in drug exposure in cohorts based on HPV status, PD-L1 expression, or treatment regimen.

### Safety

None of the patients experienced a DLT at the planned maximum administered doses, and the maximum tolerated dose (MTD) for this combination was not determined. All patients had at least one AE of any cause. Treatment-related AEs (TRAEs) of any grade occurred in 49 patients (69.0%; Table 1), with the most common ones being fatigue (32.4%), diarrhea (21.1%), pruritus (19.7%), and decreased appetite (11.3%; Table 2). Grade 3/4 TRAEs occurred in 22 (31%) patients (Table 1) and included elevated lipase levels (7%), diarrhea (5.6%), fatigue (4.2%), and hyponatremia (4.2%; Supplementary Table S2, available at https://doi.org/10.1016/j.esmoop.2024.103646). The time to onset of grade 3/4 TRAEs ranged from 9 to 337 days and duration ranged from 1 to 85 days. Five grade 3/4 TRAEs led to discontinuation of durvalumab and/or tremelimumab: diarrhea (*n* = 3), elevated glucose levels (*n* = 1), pneumonitis (*n* = 1), elevated lipase levels (*n* = 1), and large intestine perforation (*n* = 1).

**Table 1:** Summary of adverse events by treatment cohort

			Dose-expansion phase			
	**Dose-exploration phase**		Previously untreated cohorts			
**AE, *n* (%)**	Q2W cohort (*n* = 3)	Q4W cohort (*n* = 6)	PD-L1 TC ≥25% (*n* = 20)	PD-L1 TC <25% (*n* = 22)	Immunotherapy-pretreated cohort(*n* = 20)	**Total population (*N* = 71)**
All-cause AEs	3 (100)	6 (100)	20 (100)	22 (100)	20 (100)	71 (100)
TRAEs	3 (100)	5 (83.3)	11 (55.0)	18 (81.8)	12 (60.0)	49 (69.0)
Grade 3/4 TRAEs^a^	2 (66.7)	3 (50.0)	5 (25.0)	6 (27.3)	6 (30.0)	22 (31.0)
Serious TRAEs	2 (66.7)	1 (16.7)	2 (10.0)	4 (18.2)	4 (20.0)	13 (18.3)
Treatment discontinuations due to TRAEs	1 (33.3)	1 (16.7)	2 (10.0)	1 (4.5)	2 (10.0)	7 (9.9)

AE, adverse event; PD-L1, programmed death-ligand 1; Q2W, every 2 weeks; Q4W, every 4 weeks; TC, tumor cell; TRAE, treatment-related adverse event.

a No grade 5 TRAEs were reported.

**Table 2:** TRAEs occurring in >5% of the total population

			Dose-expansion phase			
	**Dose-exploration phase**		Previously untreated cohorts			
**TRAE, *n* (%)**^a^	Q2W cohort (*n* = 3)	Q4W cohort (*n* = 6)	PD-L1 TC ≥25% (*n* = 20)	PD-L1 TC <25% (*n* = 22)	Immunotherapy-pretreated cohort (*n* = 20)	**Total population (*N* = 71)**
Any	3 (100)	5 (83.3)	11 (55.0)	18 (81.8)	12 (60.0)	49 (69.0)
Fatigue	1 (33.3)	1 (16.7)	5 (25.0)	9 (40.9)	7 (35.0)	23 (32.4)
Diarrhea	1 (33.3)	2 (33.3)	4 (20.0)	5 (22.7)	3 (15.0)	15 (21.1)
Pruritus	0 (0)	2 (33.3)	2 (10.0)	6 (27.3)	4 (20.0)	14 (19.7)
Decreased appetite	1 (33.3)	0 (0)	2 (10.0)	4 (18.2)	1 (5.0)	8 (11.3)
Lipase increased	0 (0)	1 (16.7)	2 (10.0)	0 (0)	4 (20.0)	7 (9.9)
Rash maculopapular	2 (66.7)	2 (33.3)	0 (0)	1 (4.5)	2 (10.0)	7 (9.9)
Hypothyroidism	0 (0)	1 (16.7)	1 (5.0)	1 (4.5)	1 (5.0)	4 (5.6)
Arthralgia	1 (33.3)	0 (0)	1 (5.0)	2 (9.1)	1 (5.0)	5 (7.0)
Dyspnea	2 (66.7)	0 (0)	0 (0)	3 (13.6)	0 (0)	5 (7.0)
Nausea	0 (0)	1 (16.7)	2 (10.0)	1 (4.5)	1 (5.0)	5 (7.0)
Pyrexia	1 (33.3)	0 (0)	0 (0)	2 (9.1)	2 (10.0)	5 (7.0)
Abdominal pain upper	0 (0)	0 (0)	2 (10.0)	2 (9.1)	0 (0)	4 (5.6)

PD-L1, programmed death-ligand 1; Q2W, every 2 weeks; Q4W, every 4 weeks; TC, tumor cell; TRAE, treatment-related adverse event.

a Patients are counted once for each preferred term regardless of the number of events.

Treatment-related AEs of special interest (AESIs) occurred in 35 (49.3%) patients (Supplementary Table S3, available at https://doi.org/10.1016/j.esmoop.2024.103646); the most common (≥5% of patients) AESIs were diarrhea (21.1%), pruritus (19.7%), rash maculopapular (9.9%), elevated lipase levels (9.9%), and hypothyroidism (5.6%).

A total of 13 (18.3%) patients had serious AEs that were considered treatment related (Table 1), with diarrhea (4.2%) being the most common. As noted above, seven patients (9.9%) discontinued due to TRAEs: diarrhea (*n* = 3), elevated glucose levels (*n* = 1), pneumonitis (*n* = 1), elevated lipase levels (*n* = 1), and large intestine perforation (*n* = 1). There were no treatment-related deaths.

### Pharmacokinetics

**Figure 1:**
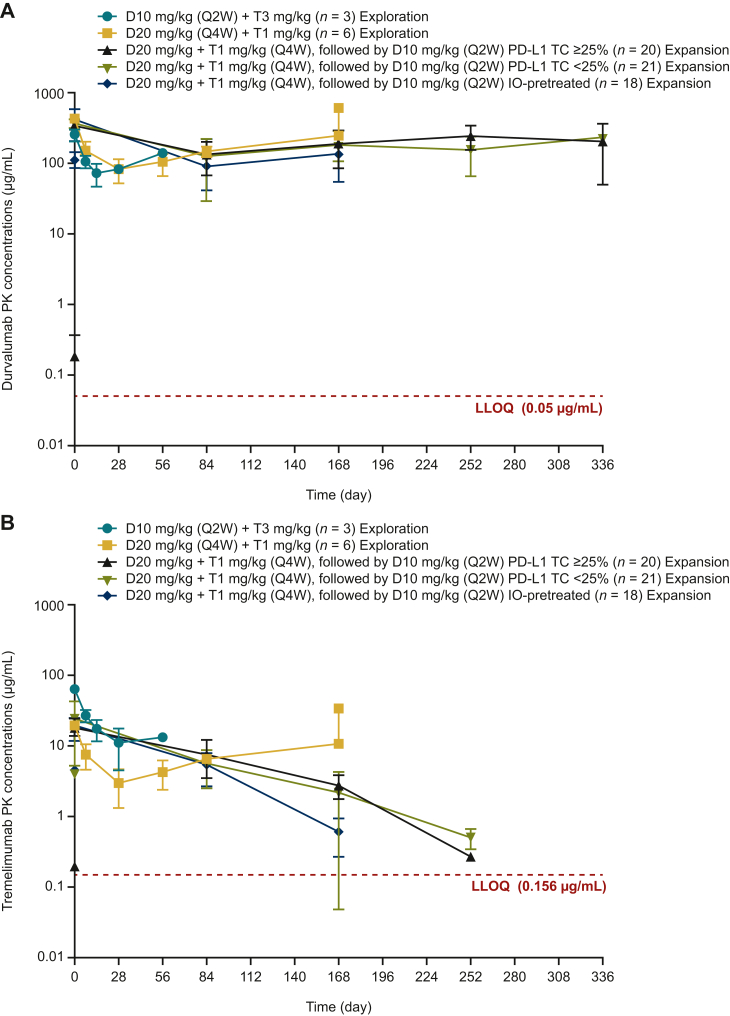
Pharmacokinetic profiles for (A) durvalumab and (B) tremelimumab in the dose-exploration and dose-expansion phases. Mean ± standard deviation concentrations at various timepoints are shown. D, durvalumab; IO, immunotherapy; LLOQ, lower limit of quantitation; PD-L1, programmed death-ligand 1; PK, pharmacokinetic; Q2W, every 2 weeks; Q4W, every 4 weeks; T, tremelimumab; TC, tumor cell.

Pharmacokinetic data were available for all 71 patients. The mean exposure profiles after repeated durvalumab and tremelimumab doses did not show evidence of drug–drug interactions (Figure 1).

### Immunogenicity

Immunogenicity data were available for 51 and 50 patients who had a valid baseline and one or more valid postbaseline ADA determinations for durvalumab and tremelimumab, respectively. The incidence of treatment-emergent (post-baseline) ADAs was 0% (0/51 patients) for durvalumab and none of the patients tested positive for neutralizing antibodies (antibodies that fully inhibit pharmacological function) at baseline or after baseline. The incidence of treatment-emergent (postbaseline) ADAs was 4% (2/50 patients) for tremelimumab and 10% (5/50) tested positive for neutralizing antibodies at baseline or after baseline. The development of ADAs did not have a clinically meaningful effect on the pharmacokinetics or safety of durvalumab or tremelimumab.

### Efficacy

For the dose-expansion cohorts, changes in tumor size over time are shown in Supplementary Figure S2, available at https://doi.org/10.1016/j.esmoop.2024.103646, and the best change in target lesion size is shown in Supplementary Figure S3, available at https://doi.org/10.1016/j.esmoop.2024.103646. In the total population, the median PFS was 1.9 months (95% CI 1.8-2.7 months) and was similar across all dose-expansion cohorts (Supplementary Figure S4A and B, available at https://doi.org/10.1016/j.esmoop.2024.103646) and dose-exploration cohorts (data not shown). The median OS in the total population was 8.6 months (95% CI 5.3-14.0 months). OS in the dose-expansion cohorts with previously untreated PD-L1 TC ≥25% and <25% and the immunotherapy-pretreated cohort is shown in Supplementary Figure S4C and D, available at https://doi.org/10.1016/j.esmoop.2024.103646, respectively. In the PD-L1 TC ≥25% group, the median OS was 5.2 months (95% CI 2.1-23.2 months) after a median follow-up of 3.4 months. In the PD-L1 TC <25% group, the median OS was 14.0 months (95% CI 5.6-19.8 months) after a median follow-up of 8.3 months; the median OS for the combined PD-L1 TC ≥25% and PD-L1 TC <25% groups was 11.0 months (95% CI 5.2-14.7 months). The median OS was 7.1 months (95% CI 4.6-10.1 months) in the immunotherapy-pretreated cohort.

The overall ORR was 5.6% (95% CI 1.6% to 13.8%); none of the patients in the dose-exploration phase had an objective response (one patient in the Q4W cohort with PD-L1 TC ≥25% had stable disease), and all four responders in the dose-expansion phase had PD-L1 TC ≥25% but no prior checkpoint inhibitor exposure (Supplementary Table S4, available at https://doi.org/10.1016/j.esmoop.2024.103646). The median time to response was 2.7 months (range 1.8-5.5 months), and the median DoR was not reached with a median follow-up of 3.4 months (range 0.4-24.4 months). At the end of the study period, three of the four responders had an ongoing response of ≥12 months.

Of the 35 patients with treatment-related AESIs of any grade, 3 (8.6%) had an objective response. Of the 36 patients who did not have treatment-related AESIs, 1 (2.8%) had a response. The treatment-related AESIs in responders were all grade 1-2. Most of the treatment-related AESIs in responders were also observed in nonresponders. In the three responders with treatment-related AESIs, the events began after the responses were reported in two patients and before the response was reported in one patient.

### Pharmacodynamics

**Figure 2:**
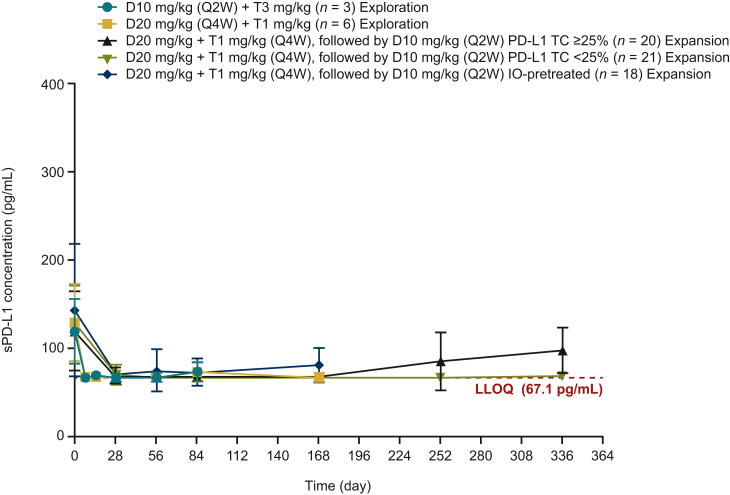
**Soluble PD-L1 concentration profiles following administration of durvalumab and tremelimumab measured by cohort**. Mean ± standard deviation concentrations at various timepoints are shown. D, durvalumab; IO, immunotherapy; LLOQ, lower limit of quantitation; PD-L1, programmed death-ligand 1; Q2W, every 2 weeks; Q4W, every 4 weeks; sPD-L1, soluble PD-L1; T, tremelimumab.

Soluble PD-L1 levels were available for all 71 patients. All cohorts showed almost complete suppression during treatment, indicating target engagement (Figure 2).

### T-cell bioanalysis

Flow cytometry results for two or more timepoints were available for 69 patients. Following treatment, the median baseline-normalized CD4+Ki67+ T cells were increased on days 8 and 15 in all cohorts and returned to near-baseline levels at most timepoints; statistically significant elevations above the median range of variability (RV) were only observed in the three dose-expansion cohorts (*P* < 0.01 by the Wilcoxon signed rank test; statistical testing was not carried out in the Q2W dose exploration cohort due to the small sample size) on days 8 and 15 (as well as on day 29 for the PD-L1 TC ≥25% cohort).

Baseline-normalized CD8+Ki67+ T cells were also observed to be elevated on day 10 or 15 in all cohorts; however, the magnitude of change on treatment was lower compared with CD4+Ki67+ T cells and statistically significant elevations above the median RV occurred only in the PD-L1 TC ≥25% cohort, on day 8 (*P* < 0.01). The magnitudes of the CD4+Ki67+ T-cell elevations were very similar in the PD-L1 TC ≥25% cohort and the PD-L1 TC <25% cohort. Modest but significant reductions below the median RV in total CD3+, CD4+, or CD8+ T cells each occurred at one or more timepoints in the immunotherapy-pretreated cohort. B-cell quantities were also reduced below the median RV in the PD-L1 TC ≥25% cohort on day 169.

## Discussion

Durvalumab plus tremelimumab was well tolerated in patients with R/M HNSCC in this study, regardless of dose or schedule. None of the patients experienced DLTs and no MTD was identified, although grade ≥3 TRAEs occurred in approximately one-third of patients. The doses ultimately selected were based on safety data from concurrent studies, including a phase Ib study in patients with NSCLC indicating that the optimal regimen was durvalumab 20 mg/kg plus tremelimumab 1 mg/kg.^14^ No pharmacokinetic evidence of drug–drug interactions was observed. Overall, the safety profile was consistent with the findings of previous early-phase studies involving durvalumab with and without tremelimumab.^10^^,^^12^, ^13^, ^14^ The number of patients with an objective response was too small to allow conclusions about a possible relationship to AESIs.

Although results were encouraging in the phase II HAWK trial of durvalumab in R/M HNSCC^10^ and other phase I/II studies involving anti-PD-(L)1 and anti-CTLA-4 combinations in various tumor types,^14^^,^^17^, ^18^, ^19^ our study showed limited clinical benefit with durvalumab plus tremelimumab. This is consistent with the phase III EAGLE study, which showed that the addition of tremelimumab to durvalumab as first-line therapy did not improve survival outcomes in patients with R/M HNSCC versus standard of care.^13^ It is also consistent with the phase III KESTREL study, which reported no significant survival benefit with durvalumab with or without tremelimumab in patients with R/M HNSCC and high PD-L1 expression.^20^ Furthermore, the phase III CheckMate 651 trial of the anti-PD-1 mAb nivolumab and the anti-CTLA-4 mAb ipilimumab for R/M HNSCC showed no significant survival benefit versus standard of care treatment, although there was a trend of OS benefit in patients with high PD-L1 expression.^21^ The CheckMate 714 study found no ORR benefit of combining ipilimumab with nivolumab versus nivolumab alone as first-line therapy in patients with R/M HNSCC.^22^

Interestingly, the current study showed a median OS of 14 months in the PD-L1 TC <25% group without any confirmed objective responses. The longer median OS compared with the PD-L1 TC ≥25% group may be due to the higher proportions of patients with ECOG PS 0 and HPV-positive disease. Thus, alternate approaches such as sequential dosing with CTLA-4 blockers before PD-L1 blockers may lead to improved immune priming compared with concurrent dosing.

Pharmacokinetic data from our study demonstrate that exposures to durvalumab and tremelimumab were not affected by coadministration. Soluble PD-L1 suppression (a surrogate for PD-L1 targeting) was observed in all cohorts regardless of durvalumab dose and concurrent exposure to tremelimumab, suggesting on-target effects.

Immunotherapy combinations are of particular interest in patients with prior exposure to anti-PD-1/PD-L1 antibodies, as it is easier to discern the effect of each investigational agent when the anti-PD-(L)1 therapy is received before rather than concurrently with another study drug as first-line treatment. In the current study, there were no responses to combination therapy in immunotherapy-pretreated patients, although two patients had sustained disease control for ≥24 weeks.

Treatment was associated with CD4+ T-cell elevation, which has been reported elsewhere as a hallmark of early response to anti-PD-L1 and anti-CTLA-4 combination therapy in patients with HNSCC.^23^

Clinical activity did not appear to be limited by target engagement, drug–drug interactions, or drug-neutralizing antibodies. A deeper understanding of the immune microenvironment and tumor–host interactions is likely key to determining the most suitable addition to PD-L1 blockade.^24^ Results from ongoing studies exploring other novel treatment combinations with PD-(L)1 inhibition, including multikinase inhibitors and anti-CD47 proteins, are highly anticipated.
