# Supplementary material for: Safety and clinical activity of durvalumab combined with tremelimumab in recurrent/metastatic head and neck squamous cell carcinoma: a multicenter phase I study
Source: ESMO Open. 2024 Jul 23;9(8):103646. doi: 10.1016/j.esmoop.2024.103646 (PMC11325272; doi:10.1016/j.esmoop.2024.103646)
Supplement: Supplementary Data [file mmc1.docx]

**Safety and Clinical Activity of Durvalumab Combined with Tremelimumab in Recurrent/Metastatic Head and Neck Squamous Cell Carcinoma:**

**A Multicenter Phase 1 Study**

Alain Algazi, Kyriakos P. Papadopoulos, Frank Tsai, Aaron R. Hansen, Natasha Angra,

Mayukh Das, Siddharth Sheth, Lillian L. Siu

**Supplementary Materials**

**Eligibility criteria**

**Entry criteria**

- Histologically or cytologically confirmed recurrent or metastatic head and neck squamous-cell carcinoma with tumors in the oral cavity, oropharynx, hypopharynx, or larynx that are incurable by local therapy
- Age ≥18 years
- Measurable disease by Response Evaluation Criteria in Solid Tumors (RECIST) version 1.1 (1), with modifications (previously irradiated lesions could be considered a target lesion if the lesion was well defined, measurable per RECIST, and had clearly progressed)
- At least one lesion suitable for biopsy
- Eastern Cooperative Oncology Group (ECOG) performance status 0 or 1
- Adequate organ and marrow function

**Exclusion criteria:**

- Active or previously documented autoimmune or inflammatory disease within the past 3 years
- Requirement for >10 mg/day prednisone or equivalent
- Prior treatment with immune-mediating therapies (apart from prior anti–PD-1, anti–PD-L1, or anti–CD-137 antibodies for patients in the dose-expansion immunotherapy-pretreated cohort, with the last dose administered ≥100 days before the planned first dose of study drug and no toxicity leading to permanent discontinuation of immunotherapy)
- Any unresolved toxicity from previous anticancer therapy

**Dose-limiting toxicities**

Dose-limiting toxicities (DLTs) were defined as any grade ≥3 toxicity that occurred during the DLT evaluation period and did not resolve quickly. Grade ≥2 pneumonitis was also considered a DLT (see below). Toxicity directly related to the primary disease or another etiology was excluded from this definition. DLTs included the following:

- Any grade 4 immune-related adverse event (irAE)
- Any grade ≥3 colitis
- Any grade 3 or 4 noninfectious pneumonitis, irrespective of duration
- Any grade ≥2 pneumonitis that does not resolve to grade ≤1 within 3 days of the initiation of maximal supportive care
- Any grade 3 irAE, excluding colitis or pneumonitis, that does not downgrade to grade 2 within 3 days after onset of the event despite optimal medical management including systemic corticosteroids or does not downgrade to grade ≤1 or baseline within 14 days
- Liver transaminase elevation >8× upper limit of normal (ULN) or total bilirubin >5× ULN
- Any grade ≥3 non-irAE, except for the exclusions listed below

The definition excludes the following conditions:

- Grade 3 fatigue for ≤7 days
- Grade 3 endocrine disorder (thyroid, pituitary, and/or adrenal insufficiency) that is managed with or without systemic corticosteroid therapy and/or hormone replacement therapy and the subject is asymptomatic
- Grade 3 inflammatory reaction attributed to a local antitumor response (e.g., inflammatory reaction at sites of metastatic disease, lymph nodes, etc.)
- Concurrent vitiligo or alopecia of any grade
- Grade 3 infusion-related reaction (first occurrence and in the absence of steroid prophylaxis) that resolves within 6 hours with appropriate clinical management
- Grade 3 or 4 neutropenia that is not associated with fever or systemic infection that improves by at least 1 grade within 3 days. Grade 3 or 4 febrile neutropenia will be a DLT regardless of duration or reversibility
- Grade 3 or 4 lymphopenia
- Grade 3 thrombocytopenia that is not associated with clinically significant bleeding that requires medical intervention and improves by at least 1 grade within 3 days
- Isolated grade 3 electrolyte abnormalities that are not associated with clinical signs or symptoms and are reversed with appropriate maximal medical intervention within 3 days

Immune-related AEs were defined as AEs of an immune nature (i.e., inflammatory) in the absence of a clear alternative etiology. In the absence of a clinically significant abnormality, repeat laboratory testing was conducted to confirm significant laboratory findings prior to designation as a DLT. Laboratory abnormalities that were not deemed to be clinically significant were not considered DLTs.

Although the rules for adjudicating DLTs in the context of dose-exploration were specified above, an AE not listed above could be defined as a DLT after consultation with the investigators, based on the emerging safety profile.

**Subgroup analyses**

**Outcomes by baseline characteristics**

To assess the consistency of treatment benefits, the objective response rate (ORR) was assessed in the total population based on baseline characteristics (age, sex, race, ECOG performance status, smoking status, tobacco use, and human papillomavirus [HPV] status). Although there were numerical differences in point estimates within subgroups, the confidence intervals (CIs) overlapped for all subgroup comparisons, and the limited sample size precluded any definitive analysis of ORR by baseline characteristics. All responding subjects were in the cohort that had PD-L1 tumor cell (TC) expression ≥25% (4 of 20 patients); of the four responders, two were HPV positive and two had an unknown HPV status; three had never smoked, and one was a current smoker.

**Outcomes by HPV status**

Subgroup analysis of progression-free survival (PFS) and overall survival (OS) by HPV status was performed for patients in the dose-expansion cohorts. Overall, in the PD-L1 TC ≥25% and PD-L1 TC <25% cohorts, median PFS was 3.6 months (95% CI, 1.8−6.8) in patients with HPV-positive status (*n* = 17), and PFS rates at 3, 6, 9, and 12 months were 52.9% (95% CI, 27.6–73.0), 29.4% (95% CI, 10.7–51.1), 11.8% (95% CI, 2.0–31.2), and 11.8% (95% CI, 2.0–31.2), respectively. In patients with HPV-negative status (*n* = 6), median PFS was 1.7 months (95% CI, 0.8−1.8) and PFS rates at 3, 6, 9, and 12 months were all 0% (95% CI, not estimable [NE]). The highest median PFS was observed in patients with PD-L1 TC ≥25% and HPV-positive status (6.8 months [95% CI, 1.9–NE]), however, sample sizes were too small to allow meaningful comparisons between cohorts. Median OS was 14.7 months (95% CI, 5.6− NE) in patients with HPV-positive status and 3.8 months (95% CI, 0.8−NE) in patients with HPV-negative status.

In the immunotherapy-pretreated cohort, median PFS was 2.0 months (95% CI, 1.2−6.4) in patients with HPV-positive status (*n* = 9) versus 2.5 months (95%, CI, 1.7−3.6) in patients with HPV-negative status (*n* = 7); median OS was 5.9 months (95% CI, 2.4−18.1) versus 7.3 months (95% CI, 4.6−10.9) respectively.

**Outcomes by PD-L1 status**

Subgroup analyses of PFS and OS by PD-L1 status were also performed in the immunotherapy-pretreated cohort. Median PFS was 3.7 months (95% CI, 2.5−7.4) in patients with PD-L1 TC ≥25% (*n* = 5) versus 1.8 months (95% CI, 1.2−3.4) in patients with PD-L1 TC <25% (*n* = 12); Median OS was 10.1 months (95% CI, 2.5−NE) and 5.0 months (95% CI, 2.4−9.0) respectively.

**Reference**

1. Eisenhauer EA, Therasse P, Bogaerts J, Schwartz LH, Sargent D, Ford R, et al. New response evaluation criteria in solid tumours: revised RECIST guideline (version 1.1). Eur J Cancer. 2009;45(2):228–47. <https://doi.org/10.1016/j.ejca.2008.10.026>

**Supplementary Table S1.** Baseline demographics and patient and disease characteristics

| **Demographic/characteristic** | **Dose-exploration phase** | | **Dose-expansion phase** | | | **Total population**  **(*N* = 71)** |
| --- | --- | --- | --- | --- | --- | --- |
|  | **Q2W cohort**  **(*n* = 3)** | **Q4W cohort**  **(*n* = 6)** | **Previously untreated cohorts** | | **Immunotherapy-pretreated cohort**  **(*n* = 20)** |  |
|  |  |  | **PD-L1 TC ≥25%**  **(*n* = 20)** | **PD-L1 TC <25%**  **(*n* = 22)** |  |  |
| Median (range) age, years  <65 years, *n* (%)  ≥65 years, *n* (%) | 56.0 (45–61)  3 (100)  0 | 55.5 (34–65)  5 (83.3)  1 (16.7) | 60.0 (37–79)  13 (65.0)  7 (35.0) | 63.5 (42–85)  14 (63.6)  8 (36.4) | 67.0 (45–90)  7 (35.0)  13 (65.0) | 63.0 (34–90)  42 (59.2)  29 (40.8) |
| Males, *n* (%) | 2 (66.7) | 4 (66.7) | 16 (80.0) | 17 (77.3) | 19 (95.0) | 58 (81.7) |
| Race, *n* (%)  Asian  Black/African American  White  Other | 0  0  3 (100)  0 | 0  1 (16.7)  5 (83.3)  0 | 2 (10.0)  1 (5.0)  16 (80.0)  1 (5.0) | 4 (18.2)  2 (9.1)  15 (68.2)  1 (4.5) | 0  1 (5.0)  18 (90.0)  1 (5.0) | 6 (8.5)  5 (7.0)  57 (80.3)  3 (4.2) |
| ECOG performance status, *n* (%)  0  1 | 1 (33.3)  2 (66.7) | 1 (16.7)  5 (83.3) | 5 (25.0)  15 (75.0) | 9 (40.9)  13 (59.1) | 6 (30.0)  14 (70.0) | 22 (31.0)  49 (69.0) |
| Smoking history, *n* (%)  Current  Former  Never | 0  1 (33.3)  2 (66.7) | 1 (16.7)  1 (16.7)  4 (66.7) | 1 (5.0)  10 (50.0)  9 (45.0) | 0  12 (54.5)  10 (45.5) | 1 (5.0)  12 (60.0)  7 (35.0) | 3 (4.2)  36 (50.7)  32 (45.1) |
| Pack no./year, median (range) | 13.0  (13.0–13.0) | 80.0  (80.0–80.0) | 20.5  (13.0–50.0) | 35.0  (5.0–90.0) | 25.5  (0.25–150.0) | 21.0  (0.25–150.0) |
| Disease stage at study entry, *n* (%)  III  IV | 0  3 (100.0) | 0  6 (100.0) | 1 (5.0)  19 (95.0) | 0  22 (100.0) | 0  20 (100.0) | 1 (1.4)  70 (98.6) |
| HPV status, *n* (%)  Positive  Negative  Unknown | 1 (33.3)  2 (66.7)  0 | 2 (33.3)  1 (16.7)  3 (50.0) | 5 (25.0)  4 (20.0)  11 (55.0) | 12 (54.5)  2 (9.1)  8 (36.4) | 9 (45.0)  7 (35.0)  4 (20.0) | 29 (40.8)  16 (22.5)  26 (36.6) |
| PD-L1 TC expression, *n* (%)^a^  ≥25%  <25% | 0  3 (100) | 1 (20.0)  4 (80.0) | 19 (100.0)  0 | 0  22 (100) | 5 (29.4)  12 (70.6) | 25 (37.9)  41 (62.1) |
| Location of primary HNSCC tumor, *n* (%)  Oropharynx  Hypopharynx  Larynx  Oral cavity | 1 (33.3)  1 (33.3)  0  1 (33.3) | 2 (33.3)  0  1 (16.7)  3 (50.0) | 7 (35.0)  0  2 (10.0)  11 (55.0) | 11 (50.0)  2 (9.1)  5 (22.7)  4 (18.2) | 10 (50.0)  0  4 (20.0)  6 (30.0) | 31 (43.7)  3 (4.2)  12 (16.9)  25 (35.2) |
| No. of previous lines of systemic therapy for recurrent/metastatic disease, *n* (%)^b^  0  1  2  3 | 1 (33.3)  0  1 (33.3)  1 (33.3) | 2 (33.3)  4 (66.7)  0  0 | 17 (94.4)  1 (5.6)  0  0 | 18 (94.7)  0  0  1 (5.3) | 0  1 (5.0)  12 (60.0)  7 (35.0) | 38 (57.6)  6 (9.1)  13 (19.7)  9 (13.6) |

^a^For exploration Q4W cohort, *n* = 5; for previously untreated PD-L1 TC ≥25% expansion cohort, *n* = 19; for immunotherapy-pretreated expansion cohort, *n* = 18 (of whom 17 were evaluable).

^b^*n* = 18 for PD-L1 ≥25% expansion cohort; n = 19 for PD-L1 <25% group expansion cohort.

ECOG, Eastern Cooperative Oncology Group; HNSCC, head and neck squamous cell carcinoma; HPV, human papillomavirus; PD-L1, programmed cell death ligand-1; Q2W, every 2 weeks; Q4W, every 4 weeks; TC, tumor cell.

**Supplementary Table 2.** Grade 3/4 treatment-related adverse events (as-treated population)

| **TRAE**  **(by preferred term), *n* (%)** | **Dose-exploration phase** | | **Dose-expansion phase** | | | **Total population**  **(*N* = 71)** |
| --- | --- | --- | --- | --- | --- | --- |
|  | **Q2W cohort**  **(*n* = 3)** | **Q4W cohort**  **(*n* = 6)** | **Previously untreated cohorts** | | **Immunotherapy-pretreated cohort**  **(*n* = 20)** |  |
|  |  |  | **PD-L1 TC ≥25%**  **(*n* = 20)** | **PD-L1 TC <25%**  **(*n* = 22)** |  |  |
| Any | 2 (66.7) | 3 (50.0) | 5 (25.0) | 6 (27.3) | 6 (30.0) | 22 (31.0) |
| Lipase increased | 0 | 1 (16.7) | 1 (5.0) | 0 | 3 (15.0) | 5 (7.0) |
| Diarrhea | 1 (33.3) | 0 | 0 | 1 (4.5) | 2 (10.0) | 4 (5.6) |
| Hyponatremia | 1 (33.3) | 0 | 0 | 1 (4.5) | 1 (5.0) | 3 (4.2) |
| Fatigue | 0 | 0 | 1 (5.0) | 0 | 2 (10.0) | 3 (4.2) |
| Anemia | 0 | 0 | 1 (5.0) | 0 | 0 | 1 (1.4) |
| Large intestine perforation | 0 | 0 | 0 | 1 (4.5) | 0 | 1 (1.4) |
| Nausea | 0 | 1 (16.7) | 0 | 0 | 0 | 1 (1.4) |
| Face edema | 0 | 0 | 1 (5.0) | 0 | 2 (10.0) | 3 (4.2) |
| Bacteremia | 0 | 0 | 0 | 1 (4.5) | 0 | 1 (1.4) |
| Septic embolus | 0 | 0 | 0 | 1 (4.5) | 0 | 1 (1.4) |
| Subdiaphragmatic abscess | 0 | 0 | 0 | 1 (4.5) | 0 | 1 (1.4) |
| Alanine aminotransferase increased | 0 | 0 | 0 | 1 (4.5) | 0 | 1 (1.4) |
| Blood glucose increased | 0 | 1 (16.7) | 0 | 0 | 0 | 1 (1.4) |
| Lymphocyte count decreased | 0 | 0 | 0 | 0 | 1 (5.0) | 1 (1.4) |
| Hyperglycemia | 0 | 0 | 0 | 1 (4.5) | 0 | 1 (1.4) |
| Hypoalbuminemia | 0 | 0 | 0 | 1 (4.5) | 0 | 1 (1.4) |
| Muscle weakness | 0 | 0 | 0 | 1 (4.5) | 0 | 1 (1.4) |
| Optic neuritis | 0 | 0 | 0 | 1 (4.5) | 0 | 1 (1.4) |
| Syncope | 0 | 0 | 0 | 1 (4.5) | 0 | 1 (1.4) |
| Pneumonitis | 0 | 0 | 1 (5.0) | 0 | 0 | 1 (1.4) |
| Stridor | 0 | 0 | 1 (5.0) | 0 | 0 | 1 (1.4) |
| Rash maculo-papular | 1 (33.3) | 0 | 0 | 0 | 0 | 1 (1.4) |
| Hypertension | 0 | 0 | 0 | 1 (4.5) | 0 | 1 (1.4) |
| Orthostatic hypotension | 0 | 0 | 0 | 1 (4.5) | 0 | 1 (1.4) |

PD-L1, programmed cell death ligand-1; Q2W, every 2 weeks; Q4W, every 4 weeks; TC, tumor cell; TRAE, treatment-related adverse event.

**Supplementary Table 3.** Treatment-related adverse events of special interest, by preferred term

| **Treatment-related AESIs**  **(by preferred term), *n* (%)** | **Dose-exploration phase** | | **Dose-expansion phase** | | | **Total population**  **(*N* = 71)** |
| --- | --- | --- | --- | --- | --- | --- |
|  | **Q2W cohort**  **(*n* = 3)** | **Q4W cohort**  **(*n* = 6)** | **Previously untreated cohorts** | | **Immunotherapy-pretreated cohort**  **(*n* = 20)** |  |
|  |  |  | **PD-L1 TC ≥25%**  **(*n* = 20)** | **PD-L1 TC <25%**  **(*n* = 22)** |  |  |
| Any | 2 (66.7) | 3 (50.0) | 9 (45.0) | 13 (59.1) | 8 (40.0) | 35 (49.3) |
| Diarrhea | 1 (33.3) | 2 (33.3) | 4 (20.0) | 5 (22.7) | 3 (15.0) | 15 (21.1) |
| Pruritus | 0 | 2 (33.3) | 2 (10.0) | 6 (27.3) | 4 (20.0) | 14 (19.7) |
| Rash maculopapular | 2 (66.7) | 2 (33.3) | 0 | 1 (4.5) | 2 (10.0) | 7 (9.9) |
| Lipase increased | 0 | 1 (16.7) | 2 (10.0) | 0) | 4 (20.0) | 7 (9.9) |
| Hypothyroidism | 0 | 1 (16.7) | 1 (5.0) | 1 (4.5) | 1 (5.0) | 4 (5.6) |
| Dermatitis acneiform | 0 | 0 | 1 (5.0) | 1 (4.5) | 0 | 2 (2.8) |
| Aspartate aminotransferase increased | 0 | 0 | 1 (5.0) | 1 (4.5) | 0 | 2 (2.8) |
| Alanine aminotransferase increased | 0 | 0 | 1 (5.0) | 1 (4.5) | 0 | 2 (2.8) |
| Blood creatinine increased | 0 | 0 | 0 | 1 (4.5) | 1 (5.0) | 2 (2.8) |
| Adrenal insufficiency | 0 | 0 | 0 | 1 (4.5) | 0 | 1 (1.4) |
| Enterocolitis | 0 | 0 | 0 | 1 (4.5) | 0 | 1 (1.4) |
| Erythema | 0 | 0 | 0 | 0 | 1 (5.0) | 1 (1.4) |
| Tri-iodothyronine free increased | 0 | 0 | 1 (5.0) | 0 | 0 | 1 (1.4) |
| Blood TSH increased | 0 | 0 | 1 (5.0) | 0 | 0 | 1 (1.4) |
| Infusion-related reaction | 0 | 0 | 0 | 1 (4.5) | 0 | 1 (1.4) |
| Optic neuritis | 0 | 0 | 0 | 1 (4.5) | 0 | 1 (1.4) |
| Pneumonitis | 0 | 0 | 1 (5.0) | 0 | 0 | 1 (1.4) |
| Rash | 0 | 0 | 1 (5.0) | 0 | 0 | 1 (1.4) |
| Rash erythematous | 0 | 0 | 0 | 1 (4.5) | 0 | 1 (1.4) |
| Rash generalized | 0 | 0 | 0 | 0 | 1 (5.0) | 1 (1.4) |
| Amylase increased | 0 | 0 | 1 (5.0) | 0 | 0 | 1 (1.4) |

AESI, adverse event of special interest; PD-L1, programmed cell death ligand-1; TSH, thyroid stimulating hormone; Q2W, every 2 weeks; Q4W, every 4 weeks; TC, tumor cell.

**Supplementary Table S4.** Objective response as measured by RECIST 1.1 in the as-treated population

| **Measurement** | **Dose-exploration phase** | | **Dose-expansion phase** | | | **Total population**  **(*N* = 71)** |
| --- | --- | --- | --- | --- | --- | --- |
|  | **Q2W cohort**  **(*n* = 3)** | **Q4W cohort**  **(*n* = 6)** | **Previously untreated cohorts** | | **Immunotherapy-pretreated cohort**  **(*n* = 20)** |  |
|  |  |  | **PD-L1 TC ≥25%**  **(*n* = 20)** | **PD-L1 TC <25%**  **(*n* = 22)** |  |  |
| ORR, *n* (%)  [95% CI] | 0  [0–70.8] | 0  [0–45.9] | 4 (20.0)  [5.7–43.7] | 0  [0–15.4] | 0  [0–16.8] | 4 (5.6)  [1.6–13.8] |
| **Best overall response, *n* (%)** | | | | | |  |
| CR | 0 | 0 | 1 (5.0) | 0 | 0 | 1 (1.4) |
| PR | 0 | 0 | 3 (15.0) | 0 | 0 | 3 (4.2) |
| SD | 0 | 2 (33.3) | 2 (10.0) | 8 (36.4) | 9 (45.0) | 21 (29.6) |
| Unconfirmed PR | 0 | 0 | 0 | 0 | 1 (5.0) | 1 (1.4) |
| PD | 3 (100) | 3 (50.0) | 8 (40.0) | 13 (59.1) | 9 (45.0) | 36 (50.7) |
| Non-evaluable | 0 | 1 (16.7) | 6 (30.0) | 1 (4.5) | 2 (10.0) | 10 (14.1) |
| DCR24,^a^ *n* (%) [95% CI] | 0  [0–70.8] | 1 (16.7)  [0.4–64.1] | 5 (25.0)  [8.7–49.1] | 3 (13.6)  [2.9–34.9] | 2 (10.0)  [1.2–31.7] | 11 (15.5)  [8.0–26.0] |
| Median (range) duration of response, weeks | NA | NA | NR (16.1, 94.1) | NA | NA | NR (16.1, 94.1) |
| Ongoing response rate,  *n* (%) | NA | NA | 3 (75.0) | NA | NA | 3 (75.0) |

^a^DCR24 defined as CR + PR + SD lasting ≥24 weeks.

CI, confidence interval; CR, complete response; DCR24, disease control rate at 24 weeks; NA, not applicable; NR, not reached; ORR, objective response rate; PD, progressive disease; PD-L1, programmed cell death ligand-1; PR, partial response; Q2W, every 2 weeks; Q4W, every 4 weeks; RECIST v1.1, Response Evaluation Criteria in Solid Tumors version 1.1; SD, stable disease; TC, tumor cell.

**Supplementary Figure 1.** Study flow diagram. Q2W, every 2 weeks; Q4W, every 4 weeks; Q12W, every 12 weeks; PD-1, programmed cell death-1; PD-L1, programmed cell death ligand-1; R/M recurrent/metastatic; TC, tumor cell.


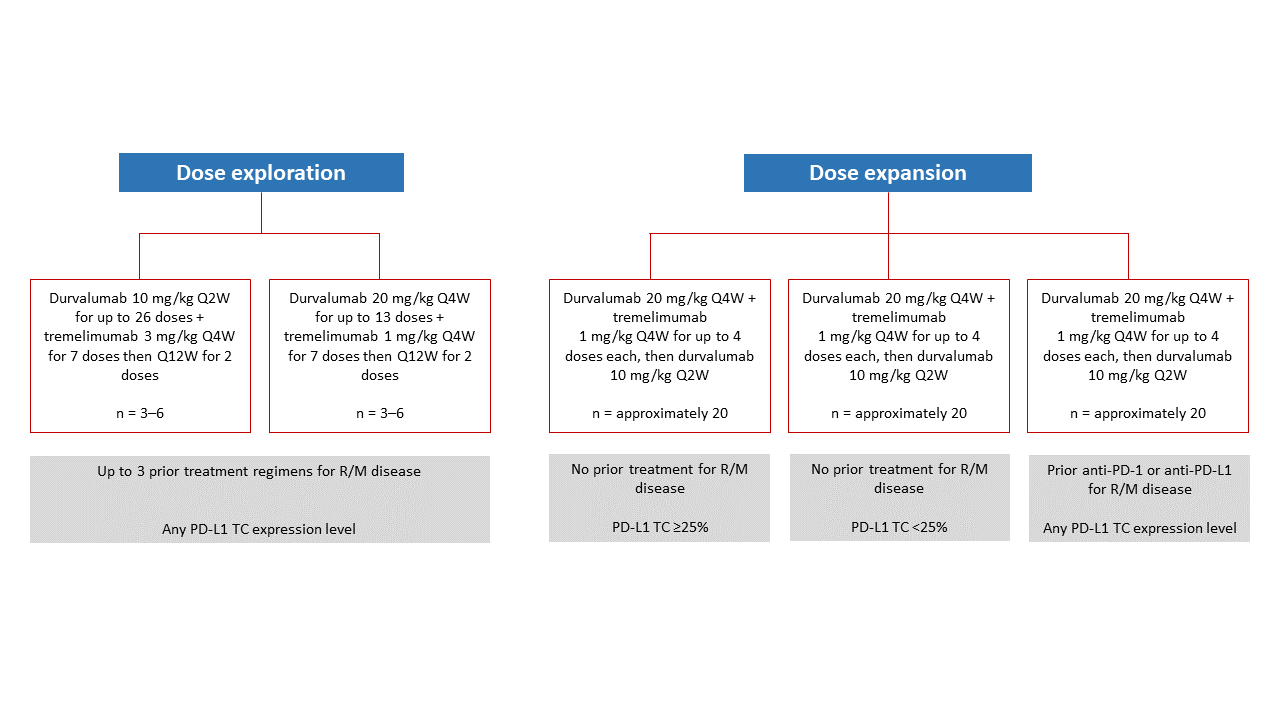


**Supplementary Figure 2.** Best change in target lesion size from baseline per investigator (RECIST v1.1) in the (A) PD-L1 TC ≥25% cohort; (B) PD-L1 TC <25% cohort; and (C) immunotherapy-pretreated cohort of the dose-expansion phase. CR, complete response; PR, partial response.

**
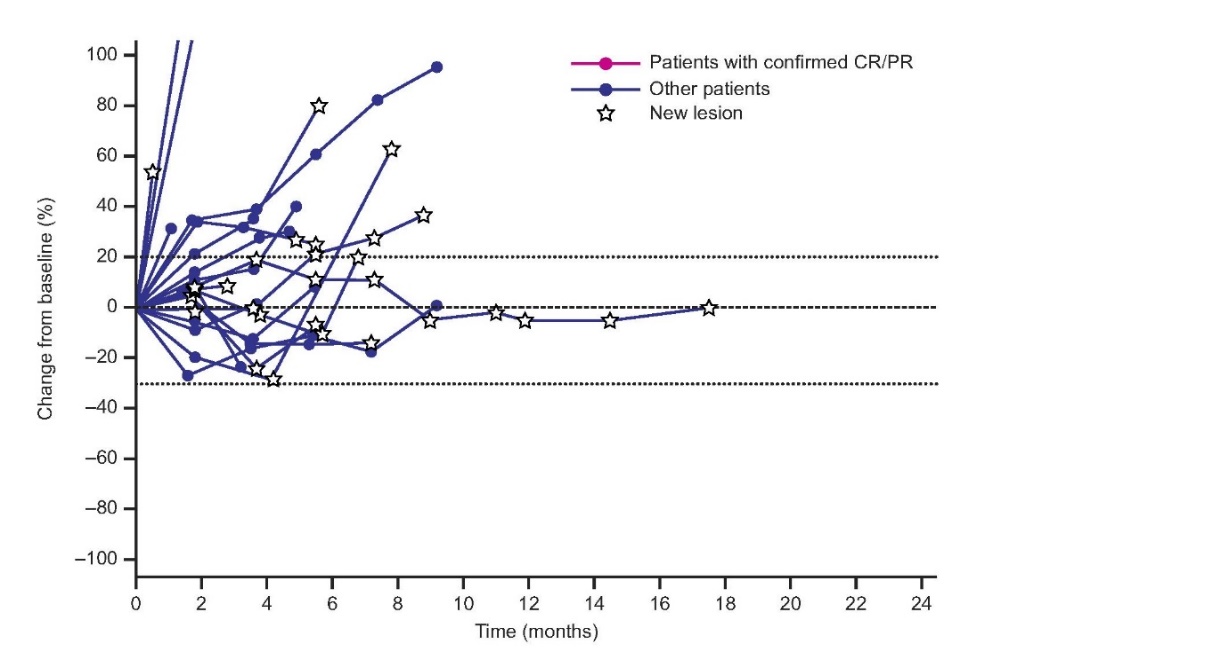
**
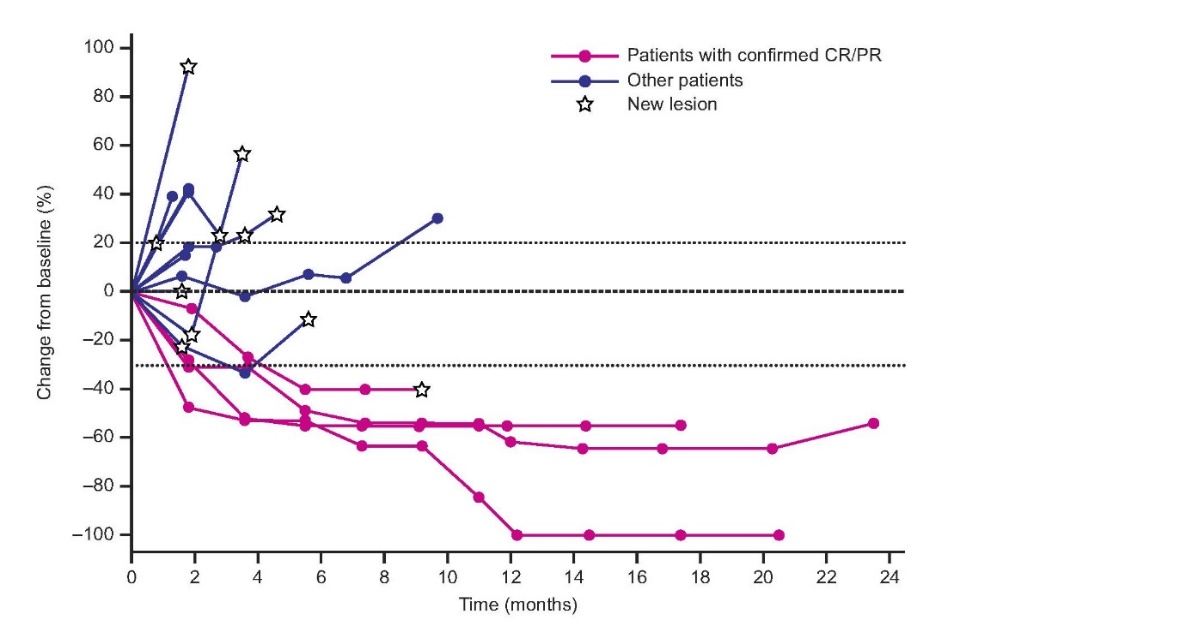
**A B**


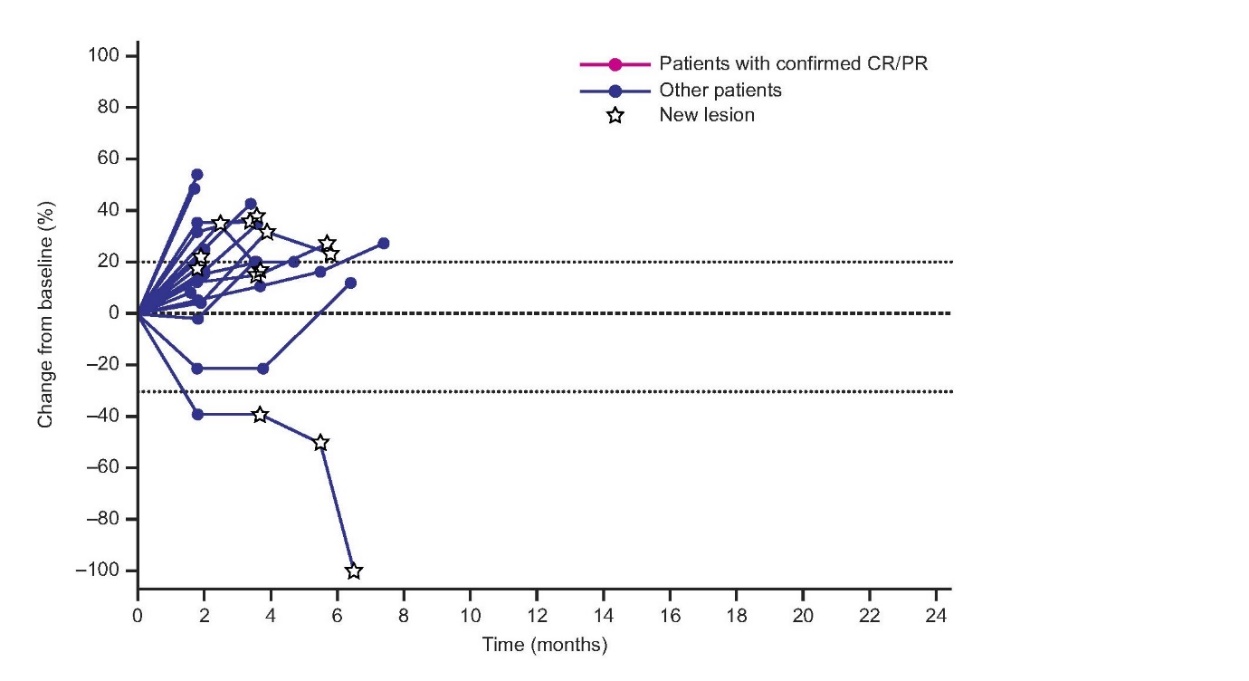
**C**

## **Supplementary Figure 3.** Best change in target lesion size from baseline per investigator (RECIST v1.1) in the (A) PD-L1 TC ≥25% cohort; (B) PD-L1 TC <25% cohort; and (C) immunotherapy-pretreated cohort of the dose-expansion phase. CR, complete response; PR, partial response.

**
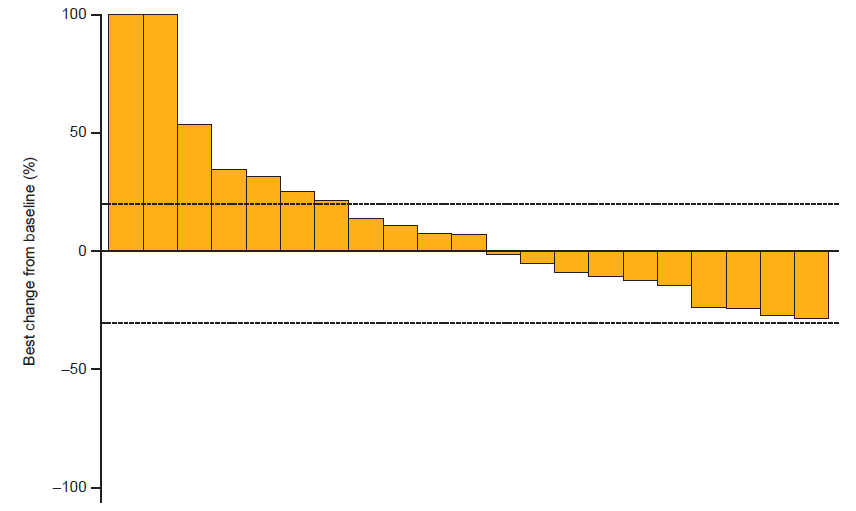
**
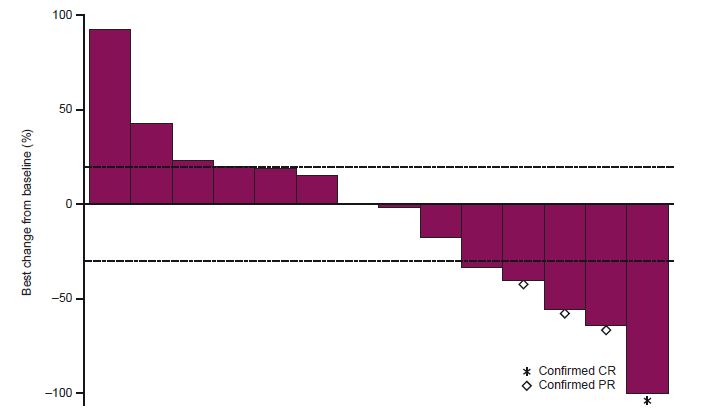
**A B**


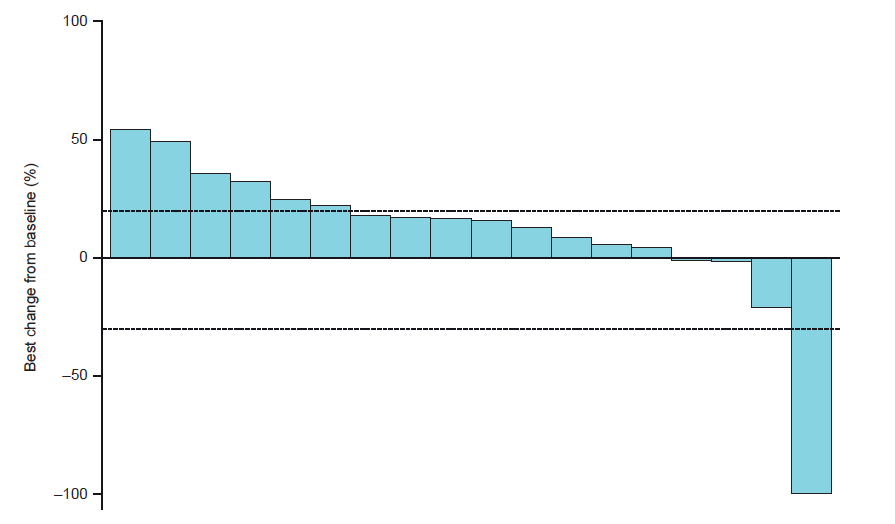
**C**

**Supplementary Figure 4.** Survival in the dose-expansion phase, by cohort. (A) Progression-free survival in the PD-L1 TC ≥25% and PD-L1 TC <25% cohorts and the overall previously untreated population; (B) progression-free survival in the immunotherapy-pretreated cohort; (C) overall survival in the PD-L1 TC ≥25% and PD-L1 TC <25% cohorts and the overall previously untreated population; (D) overall survival in the immunotherapy-pretreated cohort. CI, confidence interval; OS, overall survival; PD-L1, programmed cell death ligand-1; PFS, progression-free survival; TC, tumor cell.

**
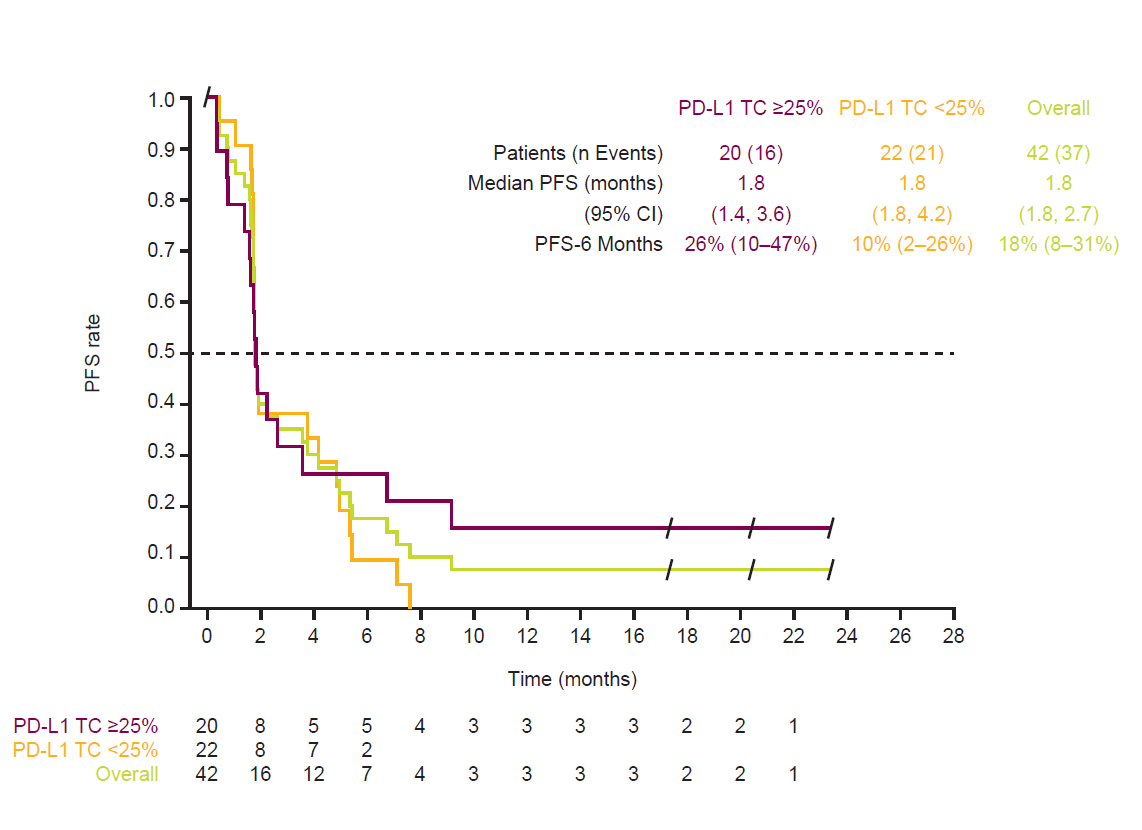
**
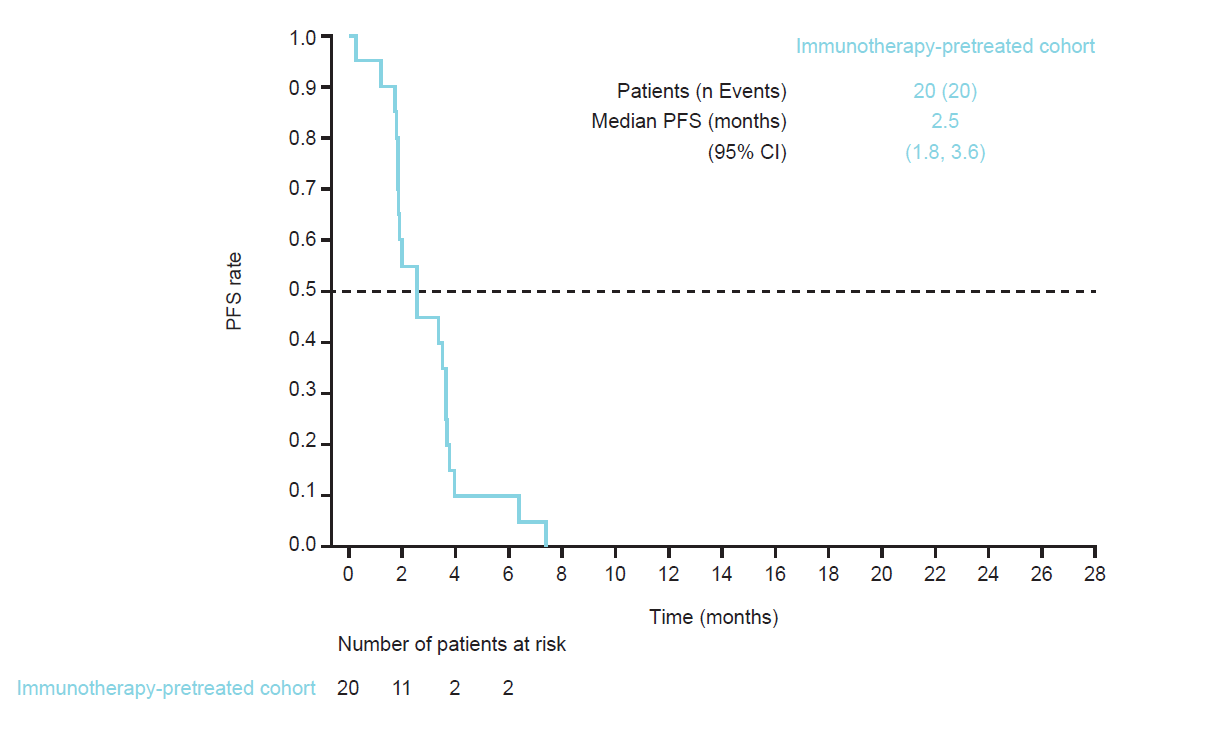
**A B**


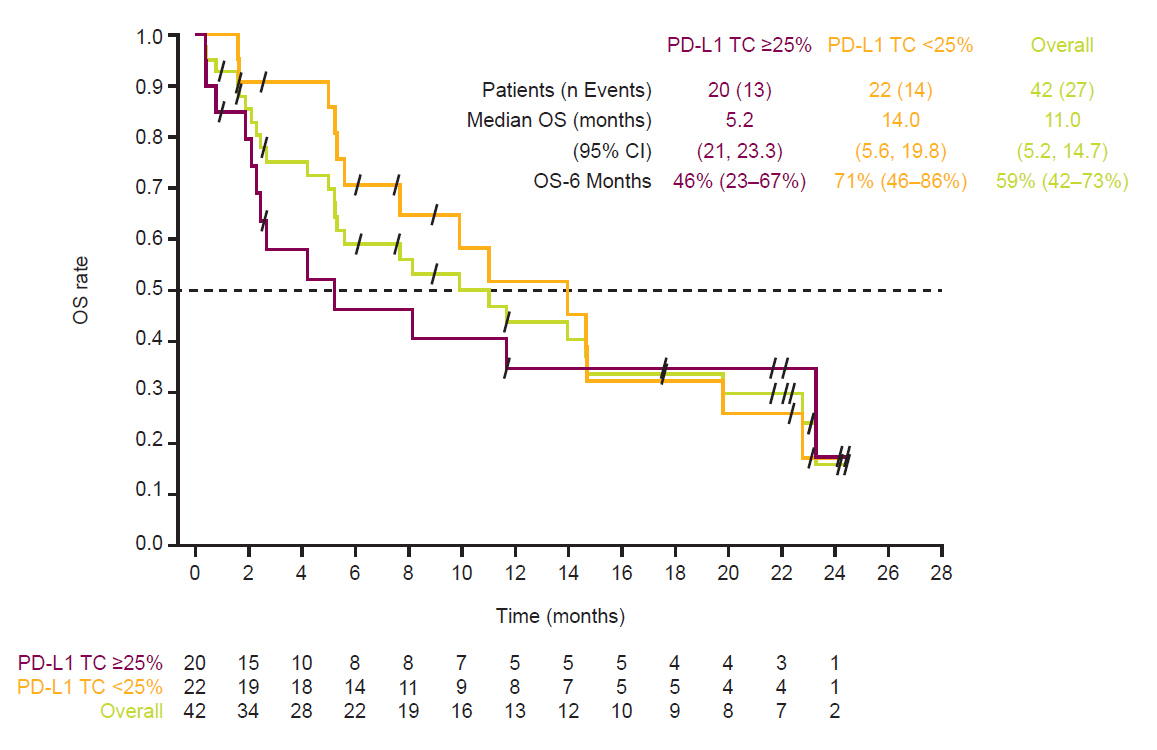
**
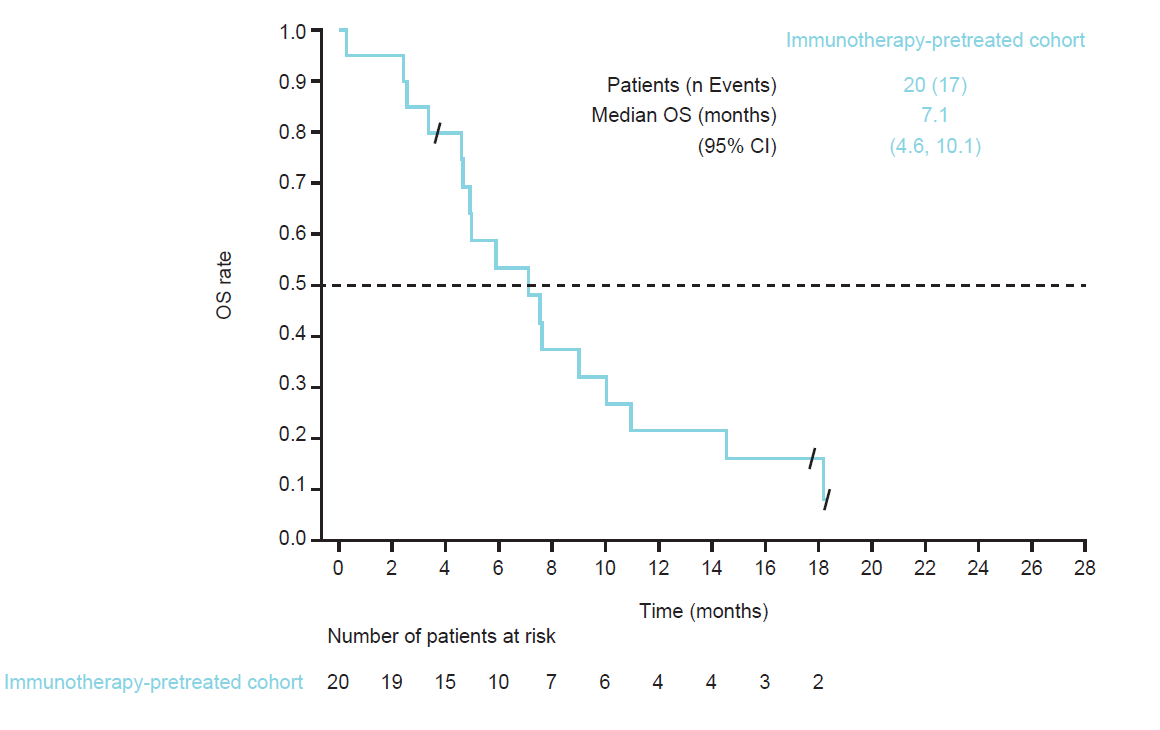
C D**
